# Species contributions to single biodiversity values under-estimate whole community contribution to a wider range of values to society

**DOI:** 10.1038/s41598-018-25339-2

**Published:** 2018-05-03

**Authors:** Matthew Hiron, Tomas Pärt, Gavin M. Siriwardena, Mark J. Whittingham

**Affiliations:** 1 School of Natural and Environmental Sciences, Newcastle University, NE1 7RU Newcastle-Upon-Tyne, UK; 2 Department of Ecology, Swedish University of Agricultural Sciences, SE-750 07 Uppsala, Sweden; 3 British Trust for Ornithology, The Nunnery, Thetford, Norfolk IP24 2PU UK

## Abstract

A major task for decision makers is deciding how to consider monetary, cultural and conservation values of biodiversity explicitly when planning sustainable land use. Thus, there is a great need to understand just what “valuing” biodiversity or species really means, e.g. regarding how many and which species are important in providing ecosystem services or other values. Constructing ecosystem-level indices, however, requires weighting the relative contribution of species to the different values. Using farmland birds, we illustrate how species contribute to different biodiversity values, namely utilitarian (pest seed predation potential), cultural (species occurrence in poetry), conservational (declines and rarity) and inherent (all species equal) value. Major contributions to each value are often made by a subset of the community and different species are important for different values, leading to no correlations or, in some cases, negative correlations between species’ relative contributions to different values. Our results and methods using relative contributions of species to biodiversity values can aid decisions when weighing different values in policies and strategies for natural resource management. We conclude that acknowledging the importance of the range of biodiversity values that are apparent from different perspectives is critical if the full value of biodiversity to society is to be realised.

## Introduction

Ecosystems are rapidly degrading and the predicted impacts of this on human well-being are profound^1^. The valuing of biodiversity for human benefit has become a central principle for policies and strategies concerning natural resource management^1–3^. Policy-makers wishing to protect nature often devise schemes based on species and therefore focus on species of conservation concern or those involved in utilitarian ecosystem services such as pollinators and enemies to crop pests^4,5^. Recent and ongoing international endeavours such as the Millennium Ecosystem Assessment (MEA)^1^ and the Intergovernmental Science-Policy Platform on Biodiversity and Ecosystem Services (IPBES)^3^ acknowledge the importance of valuing nature and biodiversity in different ways. For example, IPBES states that the process of recommending actions for maintaining biodiversity and ecosystem service relationships will “consider a whole range of values from monetary to spiritual and from instrumental to relational”^6^. However, concerns have been expressed about how to capture non-monetary values of nature and biodiversity^7^.

Why does measuring contribution of species to different values matter? The relationships between a range of single ecosystem services or ecological functions (which may or may not be of direct importance to humans) with increasing biodiversity are generally positive and non-linear and take the form of a saturating curve^8,9^, sometimes with just a few species providing a disproportionate amount of benefit. For example, an estimated 2% of insect species can provide 80% of pollination services^5^. Such a result may suggest that biodiversity management policies should focus on a few common species. If these species have a high utilitarian value and low conservation value it is then up to policy makers to decide priorities. In order to do this prioritization efficiently, with an evidence base, we need to understand a range of values or services (both monetary and non-monetary) provided by species and how these trade off. This requires quantitative information on how individual species contribute to the different values that could influence conservation policy. Here we show that scores reflecting species’ relative contribution to different biodiversity values can be compiled by using existing information and can be used to evaluate a community’s potential provision of benefits from different philosophical viewpoints.

Placing value on different aspects of nature and biodiversity is complex but is an overarching goal when using ecosystem services to motivate conservation^2,3^. The term “value”, when used for highlighting beneficial ecosystem services, or for other reasons to protect biodiversity, can include values related to markedly different areas such as economics^5,10,11^, culture, inspiration and human wellbeing^12–14^ as well as nature’s intrinsic value^7^. These types of biodiversity values have been included in general texts about conservation biology for some time^15^ and in recent international endeavours such as the Millennium Ecosystem Assessment (MEA)^1^ and IPBES^3^. Recently, Pearson^16^ suggested to group biodiversity values in three broad classes of values, namely (i) “Utilitarian value” (or instrumental value) refers to the uses humans derive from nature which are often associated with monetary valuation such as the economic value of insect pollinators for crops or organisms as consumers of pests. These biodiversity values are clearly and easily linked to many ecosystem services arguments. (ii) “Intrinsic value” (or inherent value) refers to the value of nature irrespective of function and human perspectives, needs and interests. An extension of that argument is that species’ value cannot be separated by function and are valued equally. (iii) “Non-use value” from Pearson^16^ refers to the value of nature to humans even when there is no direct use. Such value has no clear monetary value. Still, “non-use values” are important from human perspectives such as inspiration for arts, desire or feelings of responsibility to conserve rare or charismatic species or to change population trends of declining species.

We have chosen to focus on the potential of species to contribute to four quantifiable biodiversity values that represent different philosophies behind reasons to conserve species: (1) the potential value of farmland bird species as consumers of economically important pest weed seeds (an utilitarian value); (2) farmland bird species as sources of inspiration to artistic impression quantified as mentions in poetry in an extensive online database (a cultural non-use value); as well as (3) and (4) degree of rarity and population trends (i.e. two traditional conservation motivators); in addition to the four values above, we also consider intrinsic value, to which all species contribute equally. The idea is to cover the three major classes of values sensu ref.^16^ and not to investigate the potential contribution of all potential values. Therefore, the framework that we present here is intended to exemplify how to capture quantitative measurements pertaining to species’ relative contribution potential to both monetary and non-monetary values of biodiversity (which we hereafter refer to as biodiversity values) and how these may trade off against one another. We acknowledge that the term “value” is troublesome to define and has many connotations. Here we use the term biodiversity value to refer to a measurement of species characteristics (considered at either species or community level) that can be linked to human benefit and/or be used for arguments for the need to conserve biodiversity. We used farmland birds as a model taxon because they are a well-known, high profile group of organisms used as an indicator to signify sustainable development (e.g. https://www.gov.uk/government/statistics/sustainable-development-indicators-sdis). Thus, using the UK farmland bird community we: (i) illustrate quantitatively how to measure and to visualize species’ relative contributions to four types of biodiversity values, namely utilitarian (predation of economically important pest weed seeds), cultural (species occurrence in poetry), conservational (declines and rarity) and intrinsic (all species equal) values; (ii) explore whether species contribute evenly to a single biodiversity value or whether a few species dominate; (iii) investigate the impacts upon different biodiversity values of the same set of species, such as asking whether single species generally score highly for more than one value, or score highly for one value index and lowly for another, and (iv) illustrate the change in contribution of all species when combining scores for all measured biodiversity values together. Clearly the list of biodiversity values could be made longer but our quantifications of species contributions to biodiversity values are not intended to be exhaustive; rather, they illustrate a framework for creating relative species-specific contributions to biodiversity values seen from different philosophical perspectives (see methods and Supplementary Information). The framework can be overlaid on top of existing and future ecosystem service classifications and applied at appropriate spatial scales with available information for specific contexts.

For scoring species’ contributions to one potential *utilitarian value* provided by farmland birds, **weed seed predation**, we used information on: (i) weed seeds in bird diet, (ii) economic importance of those weed species for agriculture and (iii) the abundance of each species in Britain. Thus, we estimate the potential relative economic importance of each of our focal farmland bird species for weed-seed predation in Britain. Although the actual magnitude of the weed-seed predation service by farmland birds in any specific location is not known and may not be biologically or economically significant, the general service of weed seed predation is a tractable potential service and we use it as a model to illustrate how the relative importance of species can be compared. For *intrinsic values*, we simply assume that all species have the same value scores and cannot be separated by methods of quantification^16^. For three examples of *non-use values* reflecting cultural and conservation values, we first score farmland bird species for their value as inspiration to humans through **poetry** by using search results from the Poemhunter database (Poemhunter.com), which contains over one million poems. Our underlying assumption is that the more a species name, and where appropriate generic name (e.g. crow), is referred to in poems, the greater its influence on human inspiration through a form of artistic impression. Second, we score species for their **rarity** by setting the population size of the species according to Musgrove *et al*.^17^ in relation to the population size of the most common species. Third, species are scored by the magnitude of trends and **population decline estimates between 1995 and 2014** from widespread robust monitoring carried out by the British Trust for Ornithology that are available online^18^. The latter two non-use values are standard ways of prioritizing species for conservation efforts and are also a primary focus of many conservation schemes (most all-encompassing being the IUCN and regional Red Lists). Further details on scoring and interpretation are available in the methods section and Supplementary Information.

## Results and Discussion

**Figure 1:**
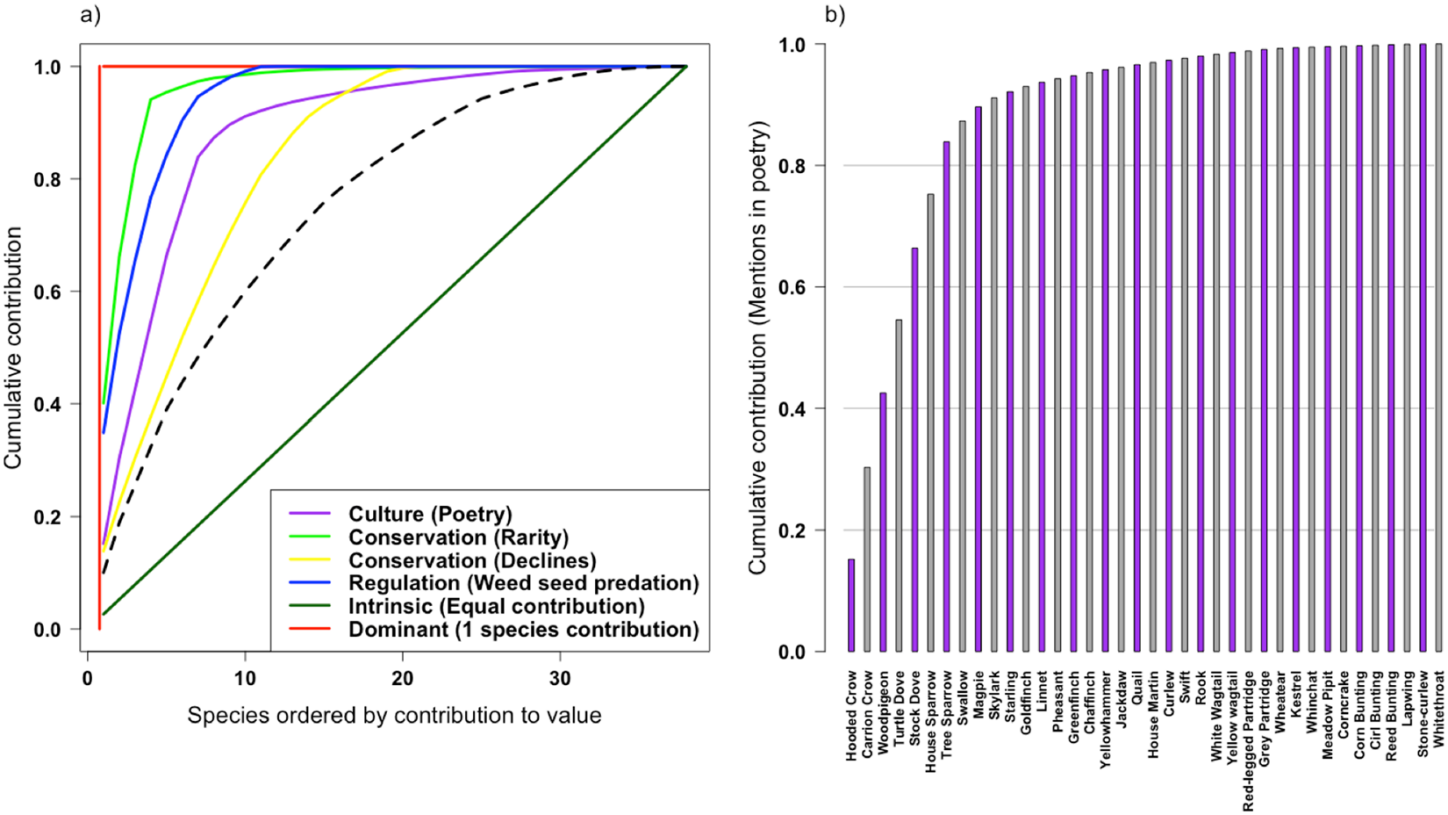
(**a**) The cumulative contribution of species to examples of biodiversity values that broadly follow ref.^16^: utilitarian (weed seed predation), intrinsic (equal contribution), and non-use (conservation and cultural). The dashed black line shows cumulative contribution when the relative contributions to scores for weed predation and poetry, declines, rarity are summed for each species. The red line illustrates a hypothetical situation where only one species contributes to a value. (**b**) Species-specific cumulative contribution to a value (in this case poetry as also illustrated with a purple curve in panel (a)). Species to the left hand side of the graphs contribute most to the measured value while those near or under the plateau region contribute little or nothing. The shape of the curve reflects details of the evenness of species contribution to measured values (see Supplementary Information Fig. 1 and text for further details).

**Figure 2:**
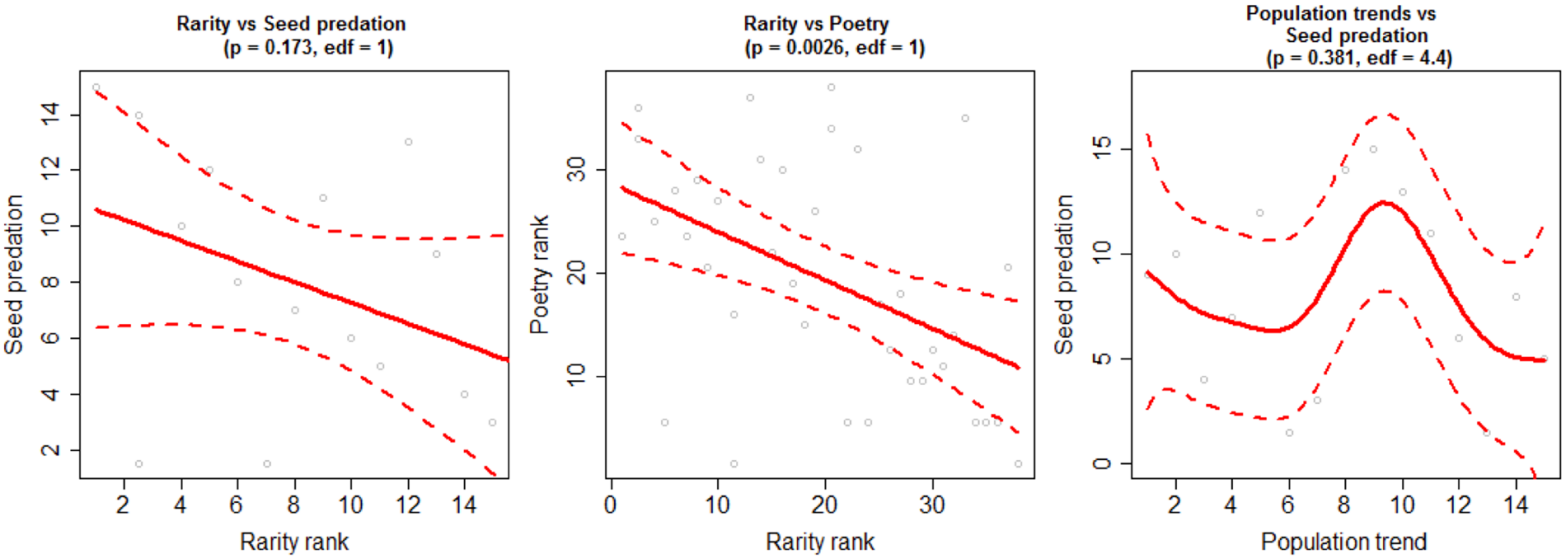
Three examples of non-parametric relationships (visualized here with Generalized Additive Models) between ranked contributions of species scores to biodiversity values. Species are ranked from common (low rank) to rare (high rank), low occurrence in poems (low rank) to high occurrence in poems (high rank), and from negative trends (low rank) to positive trends (high rank). Most bivariate relationships investigated were weak and not statistically significant. However, rare species scored low for poetry (P = 0.0026). Rare seed-eating species also tended to score lower for weed seed predation. The hump in the population trends vs seed predation figure shows that some species scoring highest for pest regulation tended to be ranked intermediately for trends (i.e. relatively stable populations or increasing populations). All bivariate relationships between measured biodiversity values are presented in the Extended Results Fig. 2. Relationships including weed seed predation are restricted to a subset including only seed-eating species.

We scored 38 farmland bird species for their relative contribution to biodiversity values, reflecting the amount of economically important weed seeds in the diet, occurrence of species name in poetry, relative rarity and population change, as described above (Extended Results Table 1). Once scored, each of the 38 species was ordered by their relative contribution and the cumulative contribution of all species to each biodiversity value was calculated. The resulting cumulative contribution curves (Fig. 1; Supplementary Information Fig. 1; Extended Results Fig. 1) show that, at the UK farmland bird community level, species’ contributions to single biodiversity value indices (not including intrinsic value) are mostly saturating at low proportions of the total species pool (see also refs^8,9^). We also ranked species from high to low contribution to each biodiversity value and investigated bivariate relationships between the values. Interestingly, species’ relative score ranks with respect to the different biodiversity values were not positively correlated (Fig. 2; Extended Results Fig. 2). That is, farmland bird species contribute at different levels to the four biodiversity values we measure. Therefore, the accumulation curve saturates less quickly when the percentage contributions of all species to all values are combined into one curve (see dashed black line in Fig. 1).

Our results suggest that looking only through the lens of single biodiversity values would underestimate the farmland bird community’s contribution more generally to ecosystem services and other biodiversity values^19–21^. This finding is in line with recent results on the relationship between biodiversity and multifunctionality that show that more species contribute to ecosystem function as more functions are considered, because functions are often species-specific^21–23^. The relationship between biodiversity and contribution to multiple values and multifunctionality will depend on the types and numbers of values/functions considered^23^. Here we only investigate a small example set of values chosen to reflect different perspectives for valuing biodiversity. Adding more values will shift the combined contribution line (Fig. 1) more towards the line of equal contribution as long as different species contribute differently to the added values. It is worth noting that we do not set thresholds of desired performance, consider levels of theoretically required functionality^21,24^ or weight the different values for their importance. Further, choices of values and representative indices, as well as of targets for performance, are subjective decisions to be made collectively by human society and, subsequently, to be framed as policies. However, our conceptual method for scoring species to reflect contribution to biodiversity values can be developed to include both thresholds and context specific weighting of different values that can then be applied to spatially or temporally explicit biodiversity data.

Our study is one of the first to score specific species objectively across, and to evaluate their relative contribution to, all three potential philosophies of valuing nature and biodiversity^16^. We show that species’ relative contributions to such different biodiversity values can be quantified (Fig. 1; Supplementary Information; Extended Results Fig. 1). The results can be used to investigate the number and identity of species contributing to specific services and values (Fig. 1; Supplementary Information Fig. 1; Extended Data Fig. 3; Extended Results Table 1) as well as potential synergies or trade-offs between multiple values (Fig. 2 and Extended Results Fig. 2). In our system (using a species group with strong ties to policy, conservation and recreation), we find little to suggest that species that are more prominent in providing one value also contribute strongly to other values. Thus, policy initiatives intended to promote biodiversity protection should be cautious when using measures of single ecosystem services to motivate nature conservation or to evaluate its success.

Given the increasing prevalence of accessible big data sources (e.g. on the world wide web), there are numerous ways that cultural values of wildlife could be measured quantitatively in addition to those we have explored (for example, the number of times species or broader natural names are mentioned across databases could be used in numerous ways to explore cultural value – e.g. street names, literature, paintings, films, religion, clothing, music, urban and rural planning environments, martial art positions, or metaphors/sayings in language). These broader ways of valuing nature could therefore be quantified and accounted for in current and future assessments attempting to value nature by looking at species contributions. It is then up to policymakers and decision makers to weigh the different values against each other in order to reach their biodiversity management goals. The more the views of different stakeholders are taken into account in such decisions, the more actions will reflect the values of biodiversity from broader society. We contend that, as shown here, that different biodiversity values can be assessed and made more transparent, easier to scrutinize and used for weighing values against each other in decision processes. Far more diverse values of biodiversity can be accounted for than is currently common practice.

## Methods

### Focal species: farmland birds

We use farmland birds as our focal community to illustrate our paper as they are a well-known species group in Europe and beyond (e.g. Web of Science search on 27/04/2016 using search term ‘Farmland birds’ resulted in 3230 articles) and their role as ecosystem service providers in farmland (particularly in Europe) has received less attention in temperate regions than other prominent groups (e.g. pollinators). The group contains species popular among the general public and that have been extensively studied by scientists, as well as species that are known to eat agricultural pests^25^. In addition, many species in this group have experienced substantial population declines^26^, in a large part due to modernization of agriculture. Thus, farmland birds as a group are potentially key flagship species for culture, recreation, scientific investigation, pest regulation and conservation. Their familiarity to non-scientists also makes them a valuable group that can be used to communicate ecological concepts and potential outcomes of conservation policy. For the purpose of this study, we use an inclusive definition of farmland birds, which includes 38 species that are considered farmland specialists as well as other species of open habitats that may be regularly encountered in rural landscapes (e.g. swifts). We include species that breed in the UK as these are often the focus for conservation measures, policy and evaluation of population trends (e.g. Breeding Bird Survey and Farmland Bird Indices e.g. http://www.eea.europa.eu/data-and-maps/indicators/species-diversity/farmland-birds-uk-quality-of) and the data available for these regarding population sizes and trends. Therefore, birds that are predominantly winter visitors are excluded from the current analyses. See Extended Results Table 1 for the list of included species.

### Calculation of value scores

#### Seed pest predation

Birds are potentially important predators of agricultural pests^27^. We measure the potential contribution of farmland birds to pest regulation as the significance of economically important agricultural weed species in the diet of each species. We use information from ref.^25^ to score how important different weed species are in the diet of farmland birds during winter (when the majority of seed consumption occurs). We follow the scoring system in that paper, where a score of 1 = 2–5%, 2 = 5–10%, & 3 = >10% of weed species seed in bird species’ diets respectively. We then adjust diet scores by a 4 level (0 to 3) numeric score reflecting the economic importance, or damage potential, of each weed species (taken from the Weed encyclopedia, http://web.adas.co.uk/WeedManager/). Thus, weeds with no economic importance (i.e. not a threat to crop yield) are assigned a score of zero while those of highest economic importance (highest threat to crop yield) are assigned a score of 3. To complete the score index that reflects each bird species’ potential of consuming economically important weed species, we include species’ relative abundance (reflecting that more abundant species eat more seeds nationally) and weight (reflecting that species that are bigger eat more seeds). Relative breeding population size was estimated as: population size for species_i_/∑ population sizes for all species. This reflects the proportion of the total estimated number of bird individuals made up of a specific species.

The total score for potential weed pest reduction for each species is calculated as:

∑(*Importance of weed species in diet* × *economic importance of weed species*) × *bird weight* × *relative population size*. Winter population size may be a more suitable abundance index to use as much seed eating will occur during that season. Therefore, we also calculated seed predation potential using winter estimates (Extended Results Fig. 3). However, as winter abundance data are available for fewer species and are not as extensive as breeding abundance data, we use breeding population sizes for the main analyses to allow for comparisons between our measured values for more species of the farmland bird community.

#### Species names in poetry

Bird species were scored according to their prevalence in poetry documented at poemhunter.com. Poemhunter is an accessible online database with 1,223,953 searchable poems (last accessed June 2016). The English name of each species was entered in to the search bar at poemhunter.com and the number of search results registered as an indication of the species’ prevalence in poetry. In some cases species names were ambiguous with other words. For species with names that can mean something else (e.g. swallow) or where hits not pertaining to birds were observed in the first 10 results, a random selection of 30 poems (judged as a fit for purpose number) was evaluated to estimate the percentage of poems pertaining to the bird name in question. Thereafter, the total number of search results for that species was then adjusted by this percentage. For example, searching for swallow resulted in 261 hits and subsequent random selection of 30 hits estimated that approximately 50% pertained to birds, thus the number of hits for this species, and thus the score for poetry, would be 261 × 0.5 = 130.5. The search results from poemhunter.com were not ordered by relevance and relevant poems were found mixed with irrelevant results at all positions in the list of results. As well as searching for English species names, we also searched for generic, family or colloquial names (such as pipits, doves and crows) where deemed appropriate. This method resulted in two possible sets of species scores to use for further analysis, where one reflects only exact species names in poems (not analysed) while the other (which we consider more suitable for our purpose) not only reflects how much the exact species name (e.g. Hooded Crow) was found in poems but also adds to the score if generic names (e.g. crow) occurred in poems. The latter approach was used here for the purpose of this study, although other bespoke scoring criteria can be used if desired for specific circumstances.

#### Rarity and population declines

Rarity can be seen as a traditional motivator for species conservation. Rare species are also highly regarded by many birdwatchers and can be seen as even having recreational value (a cultural ecosystem service). We use the breeding population size for each species obtained from ref.^17^ for calculations. Population size estimates are presented in units of e.g. males, territories or pairs. We assume that each of these measures represents two individuals and adjust population sizes by multiplying with 2 to represent number of individuals (although for our calculations relative estimates will not be affected by this procedure). We calculate the relative rarity of species as maximum population size/population size of species_i_. This scores bird species along a relative rare-common gradient on a ratio scale. For example, chaffinch (the species with the largest population size) has an estimated population of 12,400,000 individuals while skylarks are estimated to have a population of 3,000,000 individuals. Thus the rarity score for chaffinch is 12,400,000/12,400,000 = 1 and for skylark is 12,400,000/3,000,000 = 4.1. Therefore, chaffinch is approx. 4.1 times more common than skylark. Population declines can also be viewed as a traditional conservation motivator. We scored species by the size of population declines reflected in the magnitude and direction of the 19 year (1995–2014) UK population changes presented in ref.^18^ (data accessed online 17 Feb 2017 at https://www.bto.org/about-birds/birdtrends/2015). We used trend estimates in two ways for analyses. First, for cumulative contributions of species to decline magnitude, species with no change or positive population change were given a score of zero as they do not contribute to the measure of this value. Second, for bivariate relationship models (Fig. 2 and Extended Results Fig. 2) both negative and positive population changes/slopes were used for ranking species as it is also of interest if species contribution to the other values relate to increasing species as well as decreasing species.

#### Calculation, explanation and rationale for accumulation curves for species contribution to the four biodiversity values

The species-specific scores for each biodiversity value described above were ordered starting with the proportional contribution of the species that contributed most to a value followed by the contribution of each subsequent species (next highest contribution and so on). This means that the cumulative relative contribution of all species to a value equals one. See Supplementary Information Fig. 1 and associated text for a detailed discussion of information that can be gained from accumulation curves.

### Statistical Analysis

Investigation of relationships between species contribution to different biodiversity values.

We used Generalised Additive Models (GAMs) to investigate bivariate relationships between ranked species contribution to biodiversity values. GAMs have the advantage of being able to handle non-linear relationships without assuming a form of relationship a priori. We used ranked scores for this analysis rather than absolute score value (used for accumulation figures) as this makes fewer assumptions about the importance of the magnitude of differences between observations when comparing different biodiversity values together. For example, the difference of rarity scores between species can be hundreds or thousands of units. While the difference between poetry scores could single or tens of units. We do not wish to imply greater relative contribution to one value because of large increments is more important than others with smaller increments (see ref.^28^) as to compare metrics they need to be on the same scale. For this reason we use more equidistant ranks as a measure to more conservatively compare biodiversity values with each other. This, in effect, simply asks whether species ranking highly for contribution to one value also rank highly for others. All GAMs were fitted using the mgcv package^29^ in R^30^.

## Electronic supplementary material

Supplementary Information
