# Supplementary material for: Species contributions to single biodiversity values under-estimate whole community contribution to a wider range of values to society
Source: Sci Rep. 2018 May 3;8:7004. doi: 10.1038/s41598-018-25339-2 (PMC5934388; doi:10.1038/s41598-018-25339-2)
Supplement: Supplementary file 1 — Supplementary Information [file 41598_2018_25339_MOESM1_ESM.pdf]

Supplementary Information

Species contributions to single biodiversity values under-estimate whole community contribution to a wider range of values to society

Matthew Hiron[ab], Tomas Pärt[b], Gavin M Siriwardena[c] & Mark J. Whittingham[a]

[a] School of Natural and Environmental Sciences, Newcastle University, NE1 7RU, UK

[b]Department of Ecology, Swedish University of Agricultural Sciences, SE-750 07 Uppsala, Sweden

[c] British Trust for Ornithology, The Nunnery, Thetford, Norfolk IP24 2PU, UK

Extended Methods

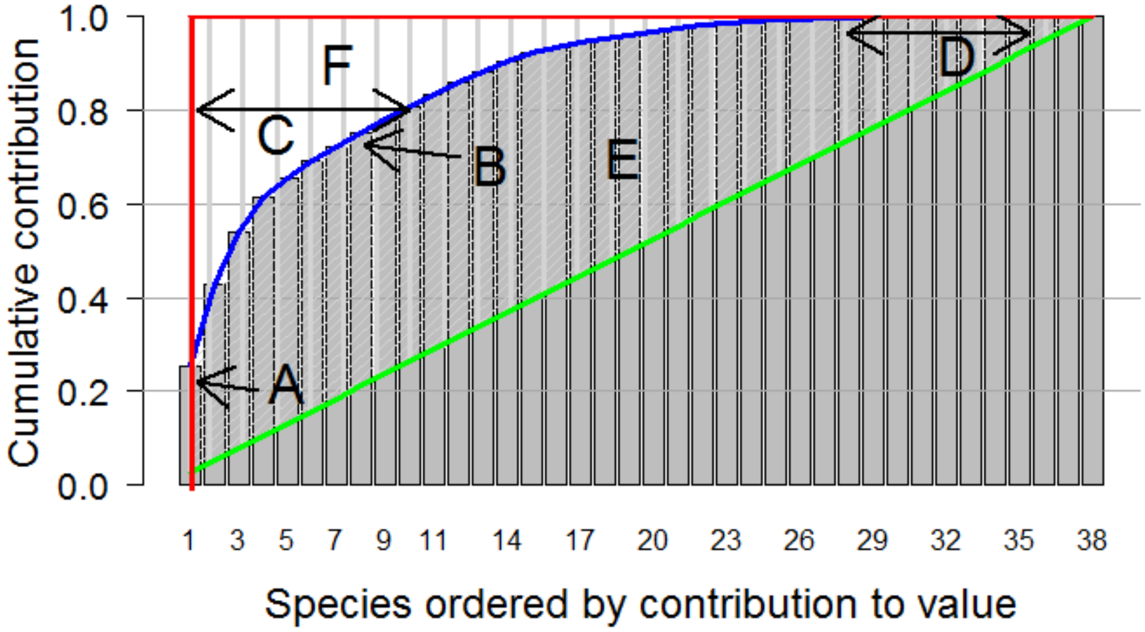

Figure. 1. The proportional cumulative contribution of species to quantified biodiversity value or ecosystem services. Information that can be interpreted from graphical representation of species´ contribution to biodiversity values. The red line shows a hypothetical extreme situation of dominance where only one species contributes to a value (Type B relationship sensu ref. 9) and the green line shows equal contribution (Type A relationship sensu ref. 9) where species in the community contribute equally. The blue line shows a (more realistic) situation intermediate between these two extremes, where a number of species contribute unevenly to a value. The shape of measured cumulative contribution curves and their relationship with hypothetical extreme scenarios can shed light on the relative ability of ecosystem service arguments to facilitate biodiversity conservation planning. The properties of curves (see below for descriptions of A-F) also describe how the species community is structured with regards to provision of biodiversity values. Where multiple values are measured, cumulative contribution curves provide a convenient way of comparing how management focus on different services will relate to biodiversity conservation and vice versa (see Fig. 1 main text)

Information gained from cumulative contribution curves

The properties of curves exemplified in Fig.1 in the main text and Fig. 1 in this section reveal how the species community is structured with regards to provision of biodiversity values or services where: **(A)** is the starting value for the species with the largest percentage contribution of the measured biodiversity value. The cumulative percent of the first few species indicates if major contribution to the biodiversity value is dominated by few species. **(B)** The incline of the slope reflects levels of ecological redundancy with a shallow slope suggesting that many species contribute similarly to a biodiversity value. This may reflect levels of resilience for a particular value if many species contribute similarly to the services

or not. In contrast, a sharp incline suggests that fewer species are required to obtain the majority of the total cumulative percentage contribution for a biodiversity value. **(C)** The number of species under the curve for a given percent indicates how many species that are needed to maintain a certain proportion of the biodiversity value (e.g. 80%). **(D)** The number of species under the curve's plateau and area of shallowest incline that shows the number of species that contribute little or nothing to a measured biodiversity value. These species might be considered superfluous for the measured value. Furthermore, different shapes of the curves will provide information on how a community is structured with regards to species' contributions to any type of value that can be relatively quantified at species level. When the species identities are presented for a particular service then species-specific contributions to measured values can also be identified. **(E)** The area between the observed cumulative contribution curve and the green line of equal contribution can be used to compare quantifiably how different services are related to biodiversity (here the number of species in the UK farmland bird community). The smaller the area between the observed cumulative contribution curve and the curve of equal contribution the greater concurrence is between a service and the number of species required to provide that service. When expressed in relation to the area between total dominant contribution and equal contribution **(F; light grey vertical shading)**, the proportion of the area under the cumulative contribution curve in relation to the area between the complete dominance and equal contribution curves becomes a useful tool to compare quantitatively how well different ecosystem services or other values relate to biodiversity.

Extended Results

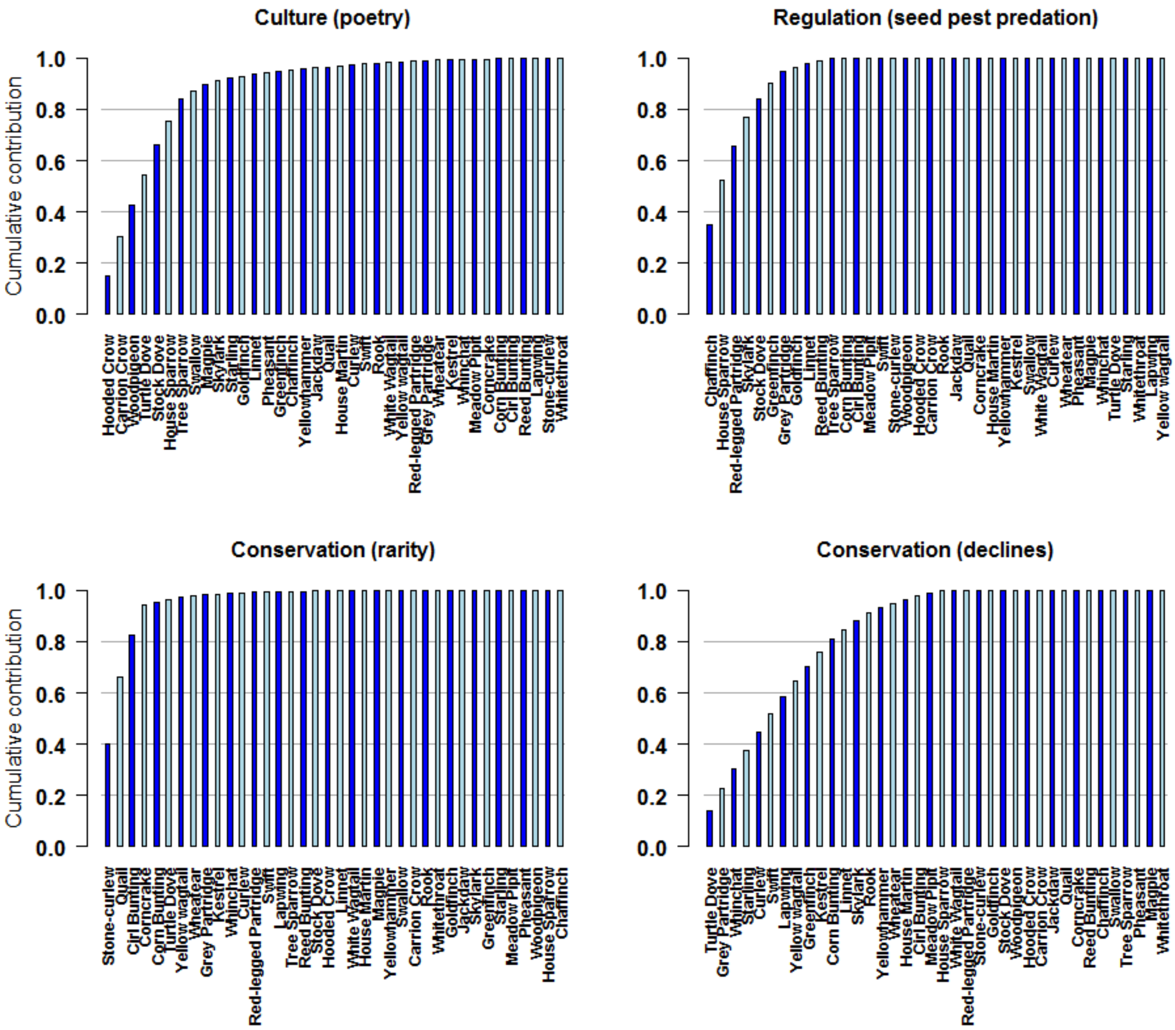

Figure. 1. Details of species specific contribution to 4 measured biodiversity values covering examples of utilitarian (weed seed predation) and non-use (conservation and cultural) values as defined by Pearson (2016)[16]. Species to the left hand side of the graph contribute most to the measured value while those near or under the plateau region contribute little or nothing. The shape of the curve reflects details about how species in a community contribute to a measured biodiversity value (see Supplementary Information extended methods Fig. 1 and associated text).

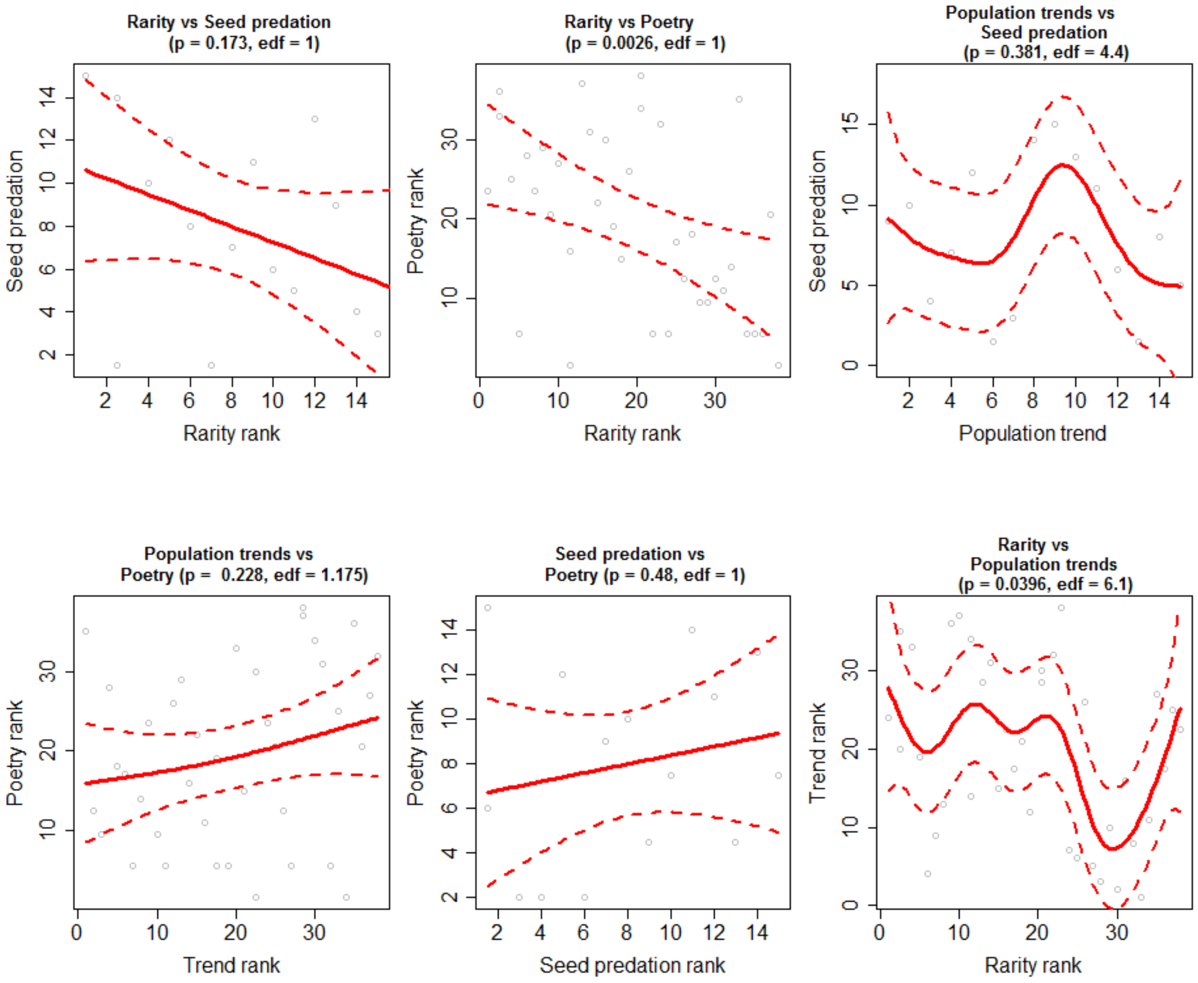

Figure. 2. Two-way non-parametric relationships between ranked contributions of species scores to biodiversity values (using Generalised Additive Models). Species are ranked from common (low rank) to rare (high rank) and from negative trends (low rank) to positive trends (high ranks). Both seed predation and poetry are ranked from lowest to highest contribution. P-values from the bivariate GAM models are shown together with the degrees of freedom (edf) for the smoother where edf = 1 corresponds to linear relationship and edf > 1 corresponds to more complex non-linear patterns.

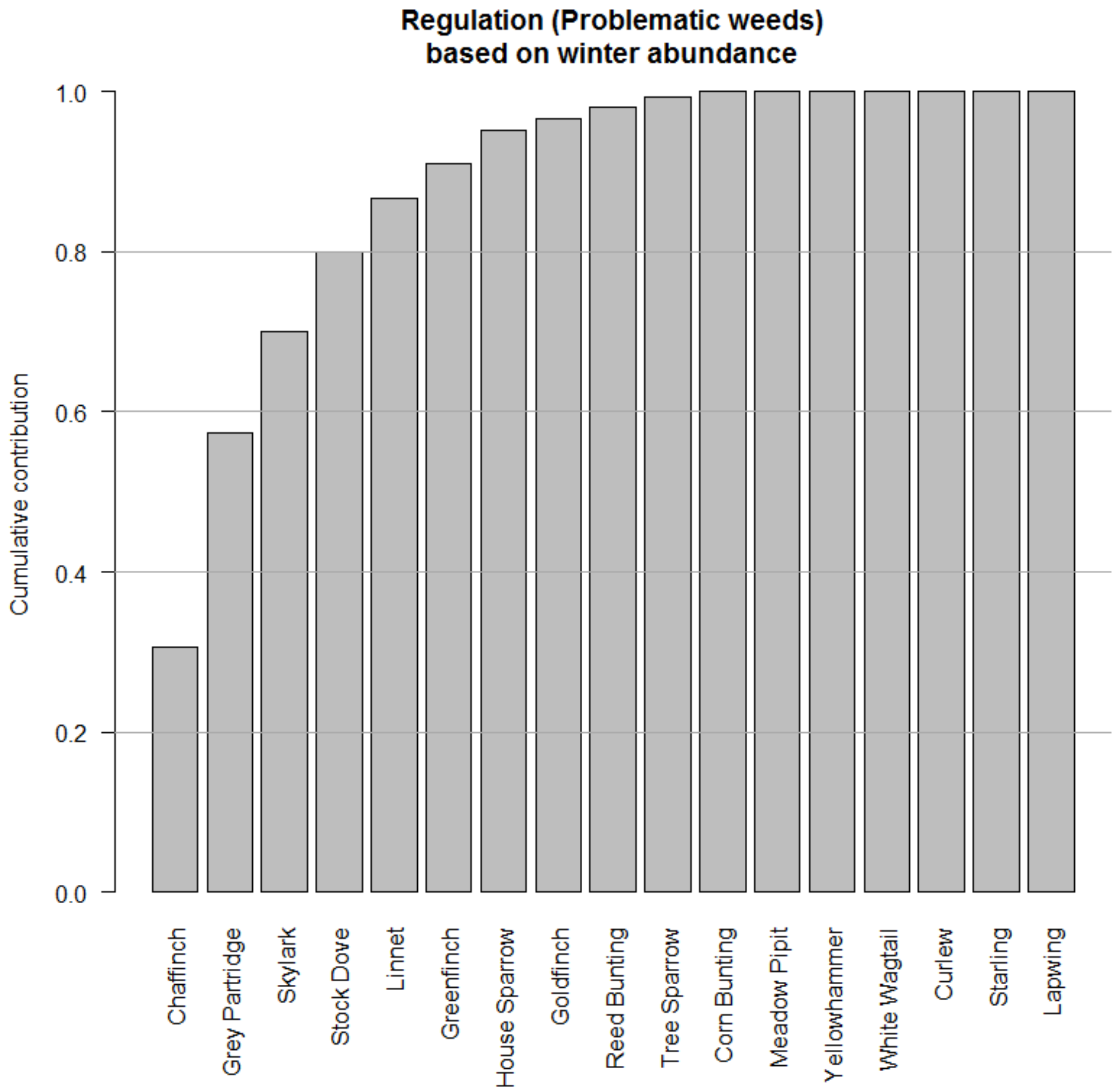

Figure. 3. The cumulative contribution of species to problematic weed seed predation calculated by using abundance estimates from the BTO's Winter Farmland Bird Survey (WFBS) data.

Table 1. The score and ranks for species specific contribution of farmland birds to poetry, weed predation, population declines/trends and rarity. Methods for scoring are described in SM methods.

Species	Poetry score	Weed predation score	Decline score	Rarity score	Trend Score	Poetry Rank	Weed predation rank	Trend rank	Rarity rank
Carrion Crow	580.5	0.0	0.0	6.2	19.0	37.0	13.0	28.5	13.0
Chaffinch	20.0	116.2	0.0	1.0	4.0	23.5	38.0	24.0	1.0
Cirl Bunting	3.0	0.0	10.0	7209.3	-10.0	5.5	26.0	17.5	36.0
Corn Bunting	3.0	0.1	34.0	563.6	-34.0	5.5	27.0	11.0	34.0
Corncrake	3.0	0.0	0.0	5166.7	14.0	5.5	13.0	27.0	35.0
Curlew	14.0	0.0	48.0	91.2	-48.0	18.0	13.0	5.0	27.0
Goldfinch	33.0	6.0	0.0	5.2	117.0	27.0	31.0	37.0	10.0
Greenfinch	20.0	20.5	39.0	3.6	-39.0	23.5	33.0	9.0	7.0
Grey Partridge	10.0	14.0	58.0	144.2	-58.0	12.5	32.0	2.0	30.0
Hooded Crow	581.5	0.0	0.0	23.8	19.0	38.0	13.0	28.5	20.5
House Martin	14.1	0.0	10.0	12.2	-10.0	19.0	13.0	17.5	17.0
House Sparrow	340.5	58.2	4.0	1.2	-4.0	33.0	37.0	20.0	2.5
Jackdaw	16.0	0.0	0.0	4.4	57.0	20.5	13.0	36.0	9.0
Kestrel	4.0	0.0	36.0	134.8	-36.0	9.5	13.0	10.0	29.0
Lapwing	3.0	0.0	43.0	44.3	-43.0	5.5	13.0	7.0	24.0
Linnet	27.0	5.3	25.0	14.4	-25.0	26.0	30.0	12.0	19.0
Magpie	90.0	0.0	0.0	10.3	0.0	30.0	13.0	22.5	16.0
Meadow Pipit	3.0	0.0	9.0	3.1	-9.0	5.5	13.0	19.0	5.0
Pheasant	22.0	0.0	0.0	2.7	32.0	25.0	13.0	33.0	4.0
Quail	16.0	0.0	0.0	11481.5	8.0	20.5	13.0	25.0	37.0
Red-legged Partridge	10.0	43.3	0.0	75.6	13.0	12.5	36.0	26.0	26.0
Reed Bunting	3.0	3.7	0.0	24.8	29.0	5.5	29.0	32.0	22.0
Rook	12.5	0.0	20.0	5.6	-20.0	16.0	13.0	14.0	11.5
Skylark	57.0	37.3	24.0	4.1	-24.0	29.0	35.0	13.0	8.0
Starling	38.0	0.0	49.0	3.3	-49.0	28.0	13.0	4.0	6.0

Stock Dove	452.8	25.2	0.0	23.8	22.0	34.0	34.0	30.0	20.5
Stone-curlew	1.0	0.0	0.0	17714.3	0.0	1.5	13.0	22.5	38.0
Swallow	130.5	0.0	0.0	7.2	26.0	31.0	13.0	31.0	14.0
Swift	13.4	0.0	47.0	71.3	-47.0	17.0	13.0	6.0	25.0
Tree Sparrow	330.5	2.7	0.0	31.0	125.0	32.0	28.0	38.0	23.0
Turtle Dove	461.8	0.0	93.0	442.9	-93.0	35.0	13.0	1.0	33.0
Wheatear	6.0	0.0	11.0	258.3	-11.0	11.0	13.0	16.0	31.0
Whinchat	4.0	0.0	53.0	131.9	-53.0	9.5	13.0	3.0	28.0
White Wagtail	12.0	0.0	2.0	13.2	-2.0	15.0	13.0	21.0	18.0
Whitethroat	1.0	0.0	0.0	5.6	33.0	1.5	13.0	34.0	11.5
Woodpigeon	466.8	0.0	0.0	1.2	36.0	36.0	13.0	35.0	2.5
Yellow wagtail	11.0	0.0	42.0	413.3	-42.0	14.0	13.0	8.0	32.0
Yellowhammer	17.0	0.0	14.0	8.7	-14.0	22.0	13.0	15.0	15.0

References to Supplementary Information and Extended Results

9 M. W. Schwartz *et al.*, Linking biodiversity to ecosystem function: implications for conservation ecology. *Oecologia* **122**, 297-305 (2000).

16 R. G. Pearson, Reasons to Conserve Nature. *Trends in Ecology & Evolution* **31**, 366-371 (2016).
